# Machine learning analysis predicts a person’s sex based on mechanical but not thermal pain thresholds

**DOI:** 10.1038/s41598-023-33337-2

**Published:** 2023-05-05

**Authors:** Jörn Lötsch, Benjamin Mayer, Dario Kringel

**Affiliations:** 1 Institute of Clinical Pharmacology, Goethe-University, Theodor-Stern-Kai 7, 60590 Frankfurt, Germany; 2 Fraunhofer Institute for Translational Medicine and Pharmacology ITMP, Theodor-Stern-Kai 7, 60596 Frankfurt, Germany

**Keywords:** Medical research, Pharmacology, Pain

## Abstract

Sex differences in pain perception have been extensively studied, but precision medicine applications such as sex-specific pain pharmacology have barely progressed beyond proof-of-concept. A data set of pain thresholds to mechanical (blunt and punctate pressure) and thermal (heat and cold) stimuli applied to non-sensitized and sensitized (capsaicin, menthol) forearm skin of 69 male and 56 female healthy volunteers was analyzed for data structures contingent with the prior sex structure using unsupervised and supervised approaches. A working hypothesis that the relevance of sex differences could be approached via reversibility of the association, i.e., sex should be identifiable from pain thresholds, was verified with trained machine learning algorithms that could infer a person's sex in a 20% validation sample not seen to the algorithms during training, with balanced accuracy of up to 79%. This was only possible with thresholds for mechanical stimuli, but not for thermal stimuli or sensitization responses, which were not sufficient to train an algorithm that could assign sex better than by guessing or when trained with nonsense (permuted) information. This enabled the translation to the molecular level of nociceptive targets that convert mechanical but not thermal information into signals interpreted as pain, which could eventually be used for pharmacological precision medicine approaches to pain. By exploiting a key feature of machine learning, which allows for the recognition of data structures and the reduction of information to the minimum relevant, experimental human pain data could be characterized in a way that incorporates "non" logic that could be translated directly to the molecular pharmacological level, pointing toward sex-specific precision medicine for pain.

## Introduction

**Figure 1:**
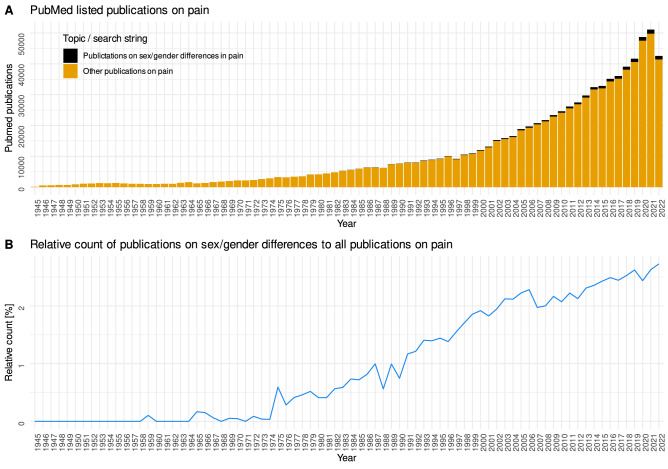
Research interests in sex aspects of pain as reflected by publication activity. Results of a computer-aided literature search of the PubMed database at https://pubmed.ncbi.nlm.nih.gov/ on November 6, 2022, using the R package "RISmed" (https://cran.r-project.org/package=RISmed^5^) and querying the string "pain AND ((sex OR gender) AND (differences OR difference)) NOT review[PT]". (**A**) Stacked bar chart of all publications listed in PubMed per year, with special emphasis on publications mentioning ”sex”/ “gender” along with "pain" compared to all publications mentioning "pain". (**B**) Line graph of relative number of publications on gender/sex differences in pain compared to all publications on pain. The figure has been created using the R software package (version 4.2.2 for Linux; http://CRAN.R-project.org/^6^) and the library “ggplot2″ (https://cran.r-project.org/package=ggplot2^7^).

Sex specific differences in pain perception have been extensively studied and reviewed^1–3^. A literature search of the PubMed database at https://pubmed.ncbi.nlm.nih.gov/ on November 6, 2022 yielded 22,322 hits, with increasing total number and relative number to publications on pain (Fig. 1). The earliest hit in this search was a paper on sex differences in the apperceptive background of pain experience published in 1959^4^, in which it was already mentioned that "females were generally predisposed to see more pain—both Intensity and duration". Thus, the sex difference in pain seems to be well studied, and the higher sensitivity of females seems to be the most common finding.

However, statistical significance does not equate with relevance. In the present analyses a first working hypothesis for addressing the relevance of observed sex differences was the reversibility of the association. If pain thresholds differ to a relevant degree between sexes, then the mutual association of pain with sex should be reversible. It should be possible to learn sex from the pain thresholds of a given sample of men and women and then generalize this skill to infer sex from the pain thresholds of new subjects. This formulation of the question of sex differences in pain is consistent with the definition of machine learning^8^. Having transferred the sex problem from statistics to machine learning, a second hypothesis arises that considers the need, typical of machine learning, to filter out the "needles" from the "haystack", i.e., to (only) use the information relevant to the problem from complex data sets. The ability to learn sex from pain thresholds may not require or be possible with every type of pain threshold, but may be (only) possible for certain types of pain thresholds. Thus, a second working hypothesis was that sex differences in pain thresholds could be used for future precision medicine approaches based on the difference in information content of thresholds for specific stimuli to infer sex. Ideally a logical "NOT" connection should be identified in the sense that sex would be related to pain threshold for one type of stimulus “but not” another. This might be taken directly to a molecular hypothesis that uses the same "NOT" link to identify a molecular sex-dependent mechanism.

These hypotheses were addressed in an available dataset^9^ containing thresholds for various noxious stimuli applied in an experimental setting to healthy young subjects. A machine learning workflow was used as described in^10^ to identify a structure in the data that reflects the sex segregation of pain thresholds and that can be used to reverse the association between sex and pain to infer a person's sex from the individual pattern of thresholds to different pain stimuli. This used algorithm building as usual in machine learning; however, the goal was not to develop an automated tool for detecting sex based on pain thresholds, but rather to use machine-learning for knowledge discovery as outlined recently^10^. Thus, the following report combines several types of machine learning methods to address the problem of sex differences in pain in a data set obtained from healthy individuals of both sexes in a human experimental setting. One goal was to identify distinctive types of pain that would provide a basis for translating the findings into a molecular context of nociceptive targets, potentially allowing sex-specific approaches to analgesic drug therapy.

## Methods

### Pain related data

The acquisition of pain-related data has been described in detail previously^9^. Briefly, the study was conducted in accordance with the Declaration of Helsinki on Biomedical Research Involving Human Subjects and was approved by the Ethics Committee of the Department of Medicine, Goethe University, Frankfurt am Main, Germany (approval number 150/11). Informed written consent was obtained from each of the participants, who were 125 unrelated healthy Caucasian volunteers (69 men, 56 women, aged 18–46 years, mean 25 ± 4.4 years) in whom pain thresholds to thermal, mechanical, and electrical stimuli were determined in nonsensitized and sensitized skin areas of the forearm in an open, nonrandomized design.

The study assessed pain thresholds defined as “the least experience of pain which a subject can recognize” (http://www.iasp-pain.org) and implemented in the study as the physical strength at which the answer to the question "does it hurt?" changes from "no" to "yes", which is why the units of pain thresholds are the units of physical strength of the applied stimuli. Thermal stimuli comprised heat and cold. Heat stimuli were applied using a 3 × 3 cm thermode (Thermal Sensory Analyzer, Medoc Advanced Medical Systems Ltd., Ramat Yishai, Israel) placed onto the skin of the left volar forearm. Its temperature was increased from 32 °C by 0.3 °C/s until the subject pressed a button at the first sensation of pain, which triggered cooling of the thermode by approximately 1.2 °C/s. Heat stimuli were applied eight times at random intervals of 25–35 s. The median of the last five responses was defined as the heat pain threshold because in previous experiments a plateau was reached after the first three measurements. Cold stimuli were administered in a similar manner, except that the temperature of the thermode decreased by 1 °C/s from 32 to 0 °C. Five repetitions were used. The cut-off temperatures of the stimulation device were 0 °C and 50 °C. Mechanical stimuli comprised blunt and punctate pressure. The former was exerted perpendicularly onto the dorsal side of mid-phalanx of the right middle finger using a pressure algometer with a circular and flat probe of 1 cm diameter (JTECH Medical, Midvale, USA). The pressure was increased at a rate of approximately 9 N/cm^2^ per second until the subject reported pain. The procedure was repeated five times every 30 s. Punctate pressure was exerted onto the left volar forearm using von Frey hairs (0.008, 0.02, 0.04, 0.07, 0.16, 0.4, 0.6, 1, 1.4, 2, 4, 6, 8, 10, 15, 26, 60, 100, 180, 300 g; North Coast Medical Inc., Morgan Hill, CA, USA). Electrical stimuli were applied using a constant current device (Neurometer^®^ CPT, Neurotron Inc., Baltimore, MD). It delivered sine-wave stimuli at 5 Hz applied via two gold electrodes placed on the medial and lateral side of the mid-phalanx of the right middle finger. Their intensity was increased from 0 to 20 mA by 0.2 mA/s until the subjects interrupted the current by releasing a button. Measurements were repeated five times every 30 s. Sensitization was assessed with punctate mechanical and heat^11^ stimuli and obtained using capsaicin cream (0.1 g, 0.1%, manufactured by the local pharmacy) applied onto a 3 × 3 cm skin area on the left volar forearm and covered with a plaster for 20 min. For cold stimuli^12^ a menthol solution (2 ml of a 40% menthol solution dissolved in ethanol) was used instead of capsaicin creme.

### Data analysis

The goal of the data analysis was to (1) identify structure in the pain threshold data reflecting the prior classification of study participants by sex, (2) to determine which variables among pain threshold drive this structure, and (3) to translate this into a molecular hypothesis for sex-dependent pain perception and treatment. A variety of analytical techniques have been applied, including artificial intelligence (AI) methods selected from its currently most widely used subfield, machine learning. Overviews of the goals and applications of machine learning in pain research have been provided elsewhere^10,13^. In the present analyses, the focus was on detecting relevant structure in the data consistent with sex segregation. This was addressed using unsupervised and supervised methods. The main difference is the prior class labeling of the cases, i.e., in unsupervised analysis, the data structure is detected without knowledge of the class structure. In supervised methods, the algorithms are given training data, i.e., variables obtained from the cases, to learn the assignment of a case to a particular class. The success of this learning process is supervised because the class labels of the training samples are known. Only later, the trained algorithms have to prove their learned skill on new cases, using the same kind of variables they were trained with to assign a case to a particular class. Perhaps a school analogy will make this clearer: during learning, the "teacher/supervisor" (researcher/programmer) supervises the learning progress of the algorithms based on the knowledge of the correct result. Only when the task has been successfully learned can the algorithm be trusted to assign the correct class label (in this case, male/female) to new cases. These were available as a 20% validation sample, separated from the data before the algorithms were trained (see below). Both classes of analysis benefit from eliminating unnecessary information from relevant content, which is a standard part of machine learning workflow referred to as feature selection^14^. In the present analysis, several methods from both classes have been applied to obtain robust and internally validated results not relying on a single method.

The programming was performed in the R language^15^ using the R software package^6^, version 4.2.2 for Linux, available from the Comprehensive R Archive Network (CRAN) at https://CRAN.R-project.org/, and in the Python language^16^ using Python version 3.8.13 for Linux, available free of charge at https://www.python.org. Analyses were performed on 1–64 cores/threads on an AMD Ryzen Threadripper 3970X (Advanced Micro Devices, Inc., Santa Clara, CA, USA) computer running Ubuntu Linux 22.04.1 LTS (Canonical, London, UK), except that deep learning neural networks were trained on an NVIDIA GeForce RTX 3060 graphics processing unit (GPU) (NVIDIA Corporation, Santa Clara, CA, USA).

### Data preprocessing

**Figure 2:**
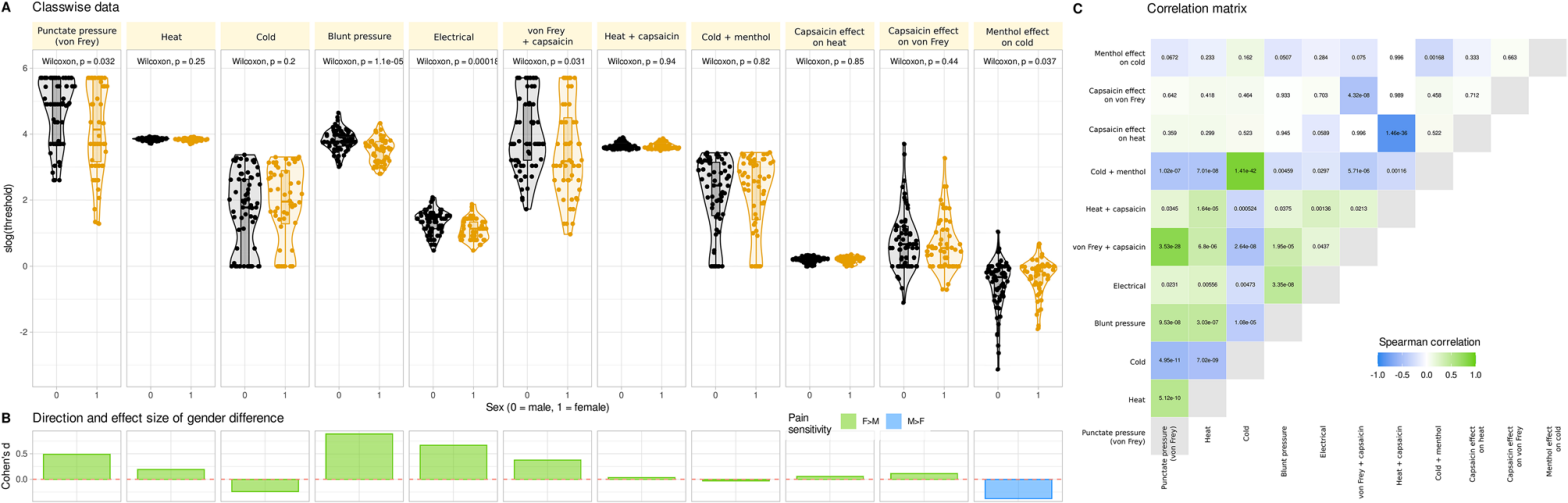
Pain thresholds data and basic statistical assessments. (**A**) Log_10_-transformed threshold to mechanical, electrical, or thermal noxious stimuli and the effects of hypersensitization by local application of capsaicin or menthol. Individual data points are presented as dots on violin plots showing the probability density distribution of the variables, overlaid with box plots where the boxes were constructed using the minimum, quartiles, median (solid line inside the box) and maximum of these values. The whiskers add 1.5 times the interquartile range (IQR) to the 75th percentile or subtract 1.5 times the IQR from the 25th percentile. At the top of the panels are the p values of the statistical comparisons for sex. (**B**) Direction and size of effect quantified using Cohen’s d. Because for cold stimuli higher threshold values mean higher pain sensitivity whereas for mechanical and heat stimuli, lower threshold values mean higher pain sensitivity, the direction of differences seems inconsistent. Therefore, the columns are colored for the direction of sex differences. (**C**) Correlation matrix of pain thresholds. Each cell is colored according to the correlation coefficient and labeled with the respective p value. The figure has been created using the R software package (version 4.2.2 for Linux; http://CRAN.R-project.org/^6^) and the R library "ggplot2" (https://cran.r-project.org/package=ggplot2^7^).

Data preprocessing consisted of log-transformation and imputation of censored data described in full detail in the supplementary materials. This provided a 125 × 11 sized input data space, *X*, of pain thresholds (125 subjects, 11 variables; Fig. 2), while the output data space, *y*, consisted of the binary sex information.

### Unsupervised analyses

Unsupervised methods were applied to analyze the univariate modal distribution of variables and cluster structures in the multivariate data space. Unsupervised methods included Gaussian mixture modeling for univariate data, five different and projection methods for multivariate data (principal component analysis (PCA)^17^, independent component analysis (ICA)^18^, multidimensional scaling (MDS)^19,20^, isomap^21^ and t-distributed stochastic neighborhood embedding (t-SNE)^22^) and seven different clustering methods (k-means clustering^23^, partitioning around medoids (PAM)^24^, hierarchical clustering with Ward's^25^, average, single, median and complete linkage). Details and results of the unsupervised analyses are reported in the supplementary materials.

Supervised analyses were performed in which machine learning algorithms were trained to infer the sex of the subjects from pain-related information. Supervised methods included five different machine learning algorithms (support vector machines (SVM)^26^, random forests^27,28^, logistic regression^29^, linear discriminant analysis (LDA)^30^ and a "deep learning"^31^ neural network^32,33^). Set-up and results of supervised analyses are described in detail as follows.

### Supervised analyses

Supervised structure detection assumed that if a computational algorithm can be trained with pain threshold data to assign a subject to the correct class, i.e., sex, so that it can infer the sex of new subjects from their pain threshold pattern, then the pain thresholds contain information that is actually relevant to the sex of the subjects.

Supervised analyses were performed using cross-validation and training/test/validation splits of the data set. Specifically, prior to feature selection and classifier training, a class-proportional random sample of 20% of the dataset was set aside as a validation sample that was not touched further during algorithm training and feature selection. The 80/20% split of the data set was done in an automated manner using our R package “opdisDownsampling” (https://cran.r-project.org/package=opdisDownsampling), which selected from 10^6^ random samples the one in which the distributions of the variables were most similar to those of these variables in the complete data set^34^. The remaining 80% of the dataset were considered as training sample, in which feature selection and classifier training were performed, using further training/test splits of this “training” sample in cross-validation settings; however, without touching the 20%-validation sample.

#### Feature selection

Feature selection aimed at filtering the "needles" from the "haystack", i.e., identifying the relevant pain threshold variables for the sex differences. Several feature selection methods were used^14^ available in the Python package “scikit-learn” (https://scikit-learn.org/stable/^35^). Univariate methods were used that are based on the false positive rate test and the family wise error rate. Multivariate feature selection methods were used that are based on calculation of an importance estimate for each variable following training of four different classification algorithms (SVM, random forests, logistic regression, LDA). After training of the algorithms, the most relevant variables were selected using methods from the "sklearn.feature_selection" module of scikit-learn. This included the F value based feature selection as implemented in the "SelectKBest" method. The number k of the features to be selected was determined by a grid search of [1,…,11] variables. Further methods comprised "SelectFromModel" (SFM), which selects features based on importance weights in the trained algorithm, recursive feature elimination (RFE), which selects features, by recursively considering smaller and smaller feature sets and generating a feature ranking, forward and backward sequential feature selection (SFS), which iteratively find the best features by adding features to a set of initially zero and all features, respectively. Furthermore, the generic permutation importance provided in the “permutation_importance” method of the “sklearn.inspection” package was used, setting the number of permutations to n_repeats = 50. The feature selection methods were applied in a 5 × 20 nested cross-validation scenario provided with the "RepeatedStratifiedKFold" method from the "sklearn.model_selection" module of "scikit-learn", setting the parameters “n_splits” = 5 and “n_repeats” = 20. All classifiers used during feature selection were tuned with respect to relevant hyperparameters.

With the two univariate methods and the six multivariate methods run with four classification algorithms, the total number of feature selection methods summed to m = 26. In the resulting 11 × 26 feature matrix, i.e., 11 pain threshold variables versus 26 selection methods, initially filled with zeros, each feature received a value of 1 when selected. This resulted in a sum score for the number of selections for each pain-related variable. The final feature set was obtained from the majority vote of the 26 selection methods. Therefore, the sum score was submitted to computed ABC (cABC) analysis^36^, an item categorization technique that divides a set of positive numerical data into three disjoint subsets labeled “A” to “C”. Subset "A" contains the "important few", which were retained as “reduced” feature sets, whereas subset C” contains the “trivial many”^37^. The Python implementation is available as our package “cABCanalysis” at https://pypi.org/project/cABCanalysis/^38^.

#### Classifier performance evaluation

The main classifiers were imported from the Python package “scikit-learn” (https://scikit-learn.org/stable/^35^). SVM, implemented as “linearSVC” and random forests were selected as two commonly used classification algorithms of different types, i.e., class separation using hyperplanes in data projected to higher dimensions, or class separation using an ensemble of simple decision trees. In addition, logistic regression and LDA were included as classical methods for class assignment. For comparison, a "deep learning"^31^ neural network^32,33^ was used to include a completely different type of classifier, using the GPU-only version of the “TensorFlow” machine learning platform (https://www.tensorflow.org^39^) for Python.

**Figure 3:**
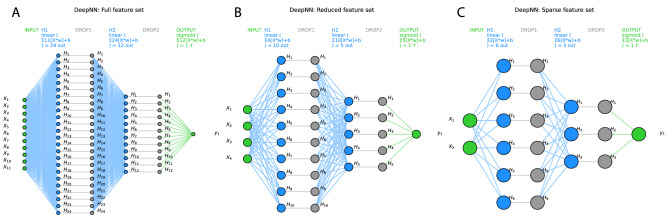
Visualization of the deep neural network architecture of the TensorFlow model used in the present analysis with (**A**) the full feature set (d = 11 pain-threshold related variables), d = 4 variables that had resulted from the feature selection steps shown in Fig. 4 as “reduced” feature set (**B**) (darker blue columns Fig. 4A), or d = 2 variables that had resulted from further narrowing the feature set to the “sparse” feature set (**C**) (darkest blue columns Fig. 4A). Variables X_1..11_ are the input variables, H_1..n_ denotes the hidden layers, and the binary output layer is shown on the right. In addition, the respective activation functions are given on the top of each layer. The graph shows the neural networks architectures as implemented with “TensorFlow” and run on an NVIDIA GeForce RTX 3060 GPU (NVIDIA Corporation, Santa Clara, CA, USA). The figure was created using Python version 3.8.13 for Linux (https://www.python.org), with the seaborn statistical data visualization package (https://seaborn.pydata.org^40^) and Python code modified from https://towardsdatascience.com/deep-learning-with-python-neural-networks-complete-tutorial-6b53c0b06af0.

The classifiers were tuned to the respective training data set using a grid search approach for different combinations of hyperparameters. For example, the tuning of the algorithms led to the selection of ridge regression as the regularization method for SVM and logistic regression, to a forest size of d = 500 trees with a maximum depth of 1 decision for random forests. For the deep neural network, after grid searches including the number of layers, number of neurons, several activation functions provided in “TensorFlow”, the number of hidden layers was set to h = 2, the number of neurons in layer 1 was set to int((n_features_ + 1)/0.5) and the number of neurons in layer 2 was set to int((n_features_ + 1)/1). A dropout layer with a rate of 0.2 was added after each layer to reduce possible overfitting. The activation functions were linear between the hidden layers and sigmoidal for the binary (men = 0, women = 1) output layer (Fig. 3). Binary crossentropy was set as the loss function, and 1000 epochs were used to train the neural network.

After feature selection, it was tested whether the selected pain threshold variables actually provided sufficient information for sex separation in a sample that was not available during classifier tuning and feature selection. Therefore, the included algorithms were trained with the full and reduced feature sets in a 5 × 20 nested cross-validation scenario, using randomly selected subsets of 67% of the original training dataset. The trained classifiers were then applied to random subsets comprising each 80% of the validation dataset that had been separated from the full original dataset prior to feature selection and classifier tuning. For the reduced feature sets, hyperparameter tuning was repeated prior to classifier performance evaluation. Balanced accuracy was used as the main parameter to evaluate the classification performance^41^. Additionally, the area under the receiver operating characteristics curve (roc-auc)^42^ was calculated. To control possible overfitting, all machine-learning algorithms were trained with pain data in that each variable was randomly permuted, with the expectation that a classifier trained with this information should not perform better than guessing, i.e., give a balanced accuracy (or a roc-auc) around 50%, else, overfitting could not be ruled out entirely. Furthermore, using a validated approach to the most informative features^43^, classifiers were also trained with the unselected features, i.e., the pain threshold variables that were kept as the result of feature selection. This provided further support that the feature selection had indeed identified the key informative variables.

### Hypothesis transfer to the level of molecular pharmacology

From the machine-learning based analysis of the sex-related information contained in pain thresholds, a molecular hypothesis for sex-dependent pain perception and treatment could be derived that narrowed the molecular background on nociceptors for mechanically but not thermally induced pain. This was addressed via knowledge retrieval from publicly accessible databases. Relevant nociceptors were identified by domain expert knowledge and classical literature search among biomedical publications in the PubMed database. The evidence on the molecular background of pain induced by mechanical stimuli provided a basis for potential translation into precision pain medicine approaches.

To transfer this in a pharmacological context, information about drugs and their molecular targets was queried from the DrugBank database^44^ at https://go.drugbank.com (version 5.1.9 dated 2022-01-04). The database was downloaded as an extensible markup language (XML) file from https://go.drugbank.com/releases/5-1-9/downloads/all-full-database. The information contained in it was processed using the R package “dbparser” (https://cran.r-project.org/package=dbparser^45^). The drug targets were available encoded as Universal Protein Resource (UniProt) (https://www.uniprot.org^46^) IDs and converted into NCBI numbers of the coding genes using the R library “org.Hs.eg.db” (https://bioconductor.org/packages/release/data/annotation/html/org.Hs.eg.db.html^47^). Approved or investigational drugs with human targets were retained.

### Ethics approval and consent to participate

The study from which the data set originates followed the Declaration of Helsinki and was approved by the Ethics Committee of Medical Faculty of the Goethe-University, Frankfurt am Main, Germany (approval number 150/11).

### Informed consent

Permission for anonymized reports of data analysis results obtained from the acquired information was included in the informed written consent.

## Results

Unsupervised analysis using five different common data projection methods and seven different common clustering algorithms ended up with a heterogeneous picture about the data structure and its correspondence to the prior classes. The respective results are reported in the supplemental information.

### Sex separation achieved with machine-learning algorithm after supervised training

#### Pain threshold variables relevant to sex segregation

**Figure 4:**
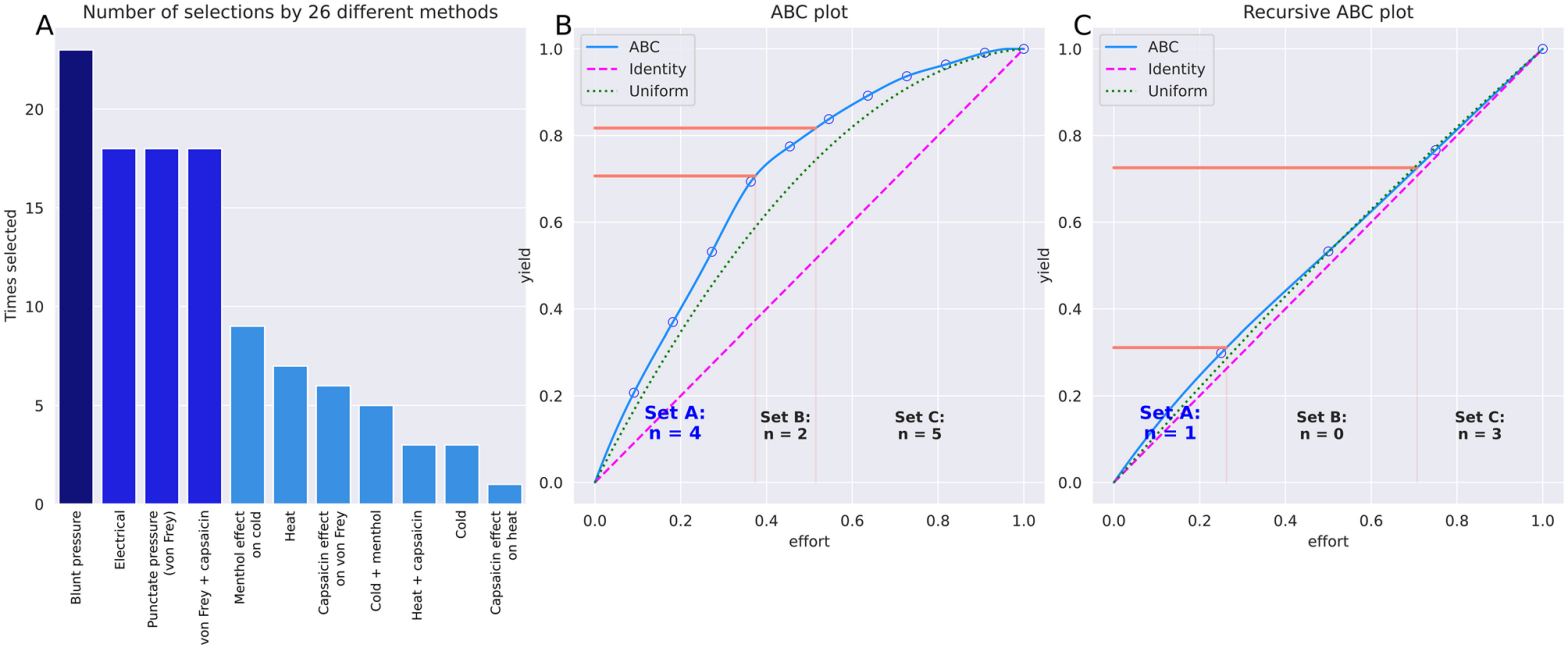
Identification of pain threshold variables that were most informative in inferring the person’ sex. (**A**) Sum scores of selections of each variable across the 26 different feature selection methods, sorted in decreasing order. The darker blue bars (left) indicate the d = 4 variables that had resulted from the feature selection as “reduced” feature set, with further narrowing to the "sparse” feature set of d = 2 variables (darker blue columns). (**B**) ABC analysis plot (blue line) showing the cumulative distribution function of the sums of occurrences in ABC category "A" in the ABC analyses previously performed with each feature selection method separately. The red lines show the boundaries between the ABC subsets "A", "B" and "C". Category "A" with d = 4 variables is considered to include the most relevant variables for class discrimination. (**C**) Result from a second cABC analysis performed on the results of the first analysis (recursive cABC analysis). The figure was created using Python version 3.8.13 for Linux (https://www.python.org), with the seaborn statistical data visualization package (https://seaborn.pydata.org^40^) and our Python package "cABCanalysis" available at https://pypi.org/project/cABCanalysis/.

Among the 26 approaches to feature selection, pain threshold for blunt pressure stimuli was selected by a majority of 22 approaches (Fig. 4). Item categorization obtained by applying cABC analysis to the sum of votes per approach identified d = 4 variables as belonging to category “A”, i.e., the “important few” according to the classical nomenclature for ABC analysis^37^, namely pain thresholds to blunt pressure, punctate pressure with and without hypersensitization by capsaicin cream, and electrical current (Fig. 4). To further narrow the focus to the most relevant variables, a recursive cABC analysis was performed, which consists of repeating the cABC analysis for the items in category “A” of a precedent cABC analysis. This provided a “sparse” feature set comprising d = 1 variable, i.e., the pain threshold to blunt pressure (Fig. 4).

#### Ability of algorithms to identify a person’s sex based on pain sensitivity

When the algorithms were trained with the full pain threshold information (d = 11 variables) from 80% of the study subjects, they identified sex in the other 20% of the subjects with a median balanced accuracy of up to 79% (Table 1). This remained almost unchanged when the algorithms were trained with the d = 4 pain thresholds of the “reduced” feature set. Even with only d = 1 pain threshold of the “sparse” feature set, i.e., pain thresholds to blunt pressure, the balanced accuracy was up to 79%.

**Table 1:** Performance of different classifier types (linear support vector machine, SVM, random forests, logistic regression, linear discriminant analysis, LDA) in assigning sex in the validation data set.

Training data set	Algorithm	Balanced accuracy	roc-auc
Full	SVM	0.6 (0.48–0.75)	0.66 (0.5–0.78)
	Random forests	0.55 (0.4–0.73)	0.58 (0.4–0.74)
	Logistic regression	0.77 (0.63–0.9)	0.77 (0.63–0.92)
	LDA	0.77 (0.62–0.89)	0.76 (0.6–0.9)
	Deep NN	0.77 (0.63–0.88)	0.77 (0.63–0.91)
Reduced	SVM	0.7 (0.55–0.79)	0.74 (0.58–0.86)
	Random forests	0.6 (0.4–0.8)	0.68 (0.47–0.82)
	Logistic regression	0.73 (0.63–0.85)	0.76 (0.63–0.91)
	LDA	0.73 (0.6–0.85)	0.76 (0.61–0.89)
	Deep NN	0.73 (0.63–0.82)	0.75 (0.67–0.86)
Sparse	SVM	0.78 (0.7–0.85)	0.76 (0.66–0.89)
	Random forests	0.74 (0.56–0.85)	0.76 (0.66–0.87)
	Logistic regression	0.78 (0.7–0.85)	0.76 (0.66–0.89)
	LDA	0.79 (0.7–0.85)	0.76 (0.66–0.89)
	Deep NN	0.75 (0.6–0.85)	0.73 (0.61–0.86)
Sparse permuted	SVM	0.5 (0.4–0.76)	0.3 (0.12–0.87)
	Random forests	0.5 (0.21–0.84)	0.46 (0.12–0.87)
	Logistic regression	0.5 (0.4–0.76)	0.3 (0.14–0.87)
	LDA	0.5 (0.4–0.76)	0.3 (0.14–0.87)
	Deep NN	0.52 (0.34–0.69)	0.52 (0.29–0.72)
Unselected features	SVM	0.5 (0.35–0.62)	0.52 (0.39–0.68)
	Random forests	0.48 (0.35–0.6)	0.41 (0.27–0.54)
	Logistic regression	0.48 (0.35–0.64)	0.49 (0.33–0.64)
	LDA	0.5 (0.35–0.63)	0.49 (0.35–0.65)
	Deep NN	0.48 (0.34–0.63)	0.5 (0.35–0.63)

The classifiers were trained in a 100-fold nested cross validation setting with subsets randomly drawn from the 80% training data set separated at the beginning of the analyses. Training was performed with all variables (d = 11 pain-threshold related variables) as “full” feature set, d = 4 variables that had resulted from the feature selection steps shown in Fig. 4 as “reduced” feature set (darker blue columns Fig. 4A), or d = 2 variables that had resulted from further narrowing the feature set to the “sparse” feature set (darkest blue columns Fig. 4A). In addition, the sex assignment task was repeated using permuted data for algorithm training or using the unselected features, i.e., the pain-threshold information that was not found to be informative for sex inference (light blue columns in Fig. 4A). The numbers are the medians of classification performance on the 20% validation data separated before feature selection and classifier training from the whole data set and not used for feature selection and algorithm training. Shown are the medians and nonparametric 95% confidence intervals (2.5th to 97.5th percentiles) from 100-fold cross-validation runs.

By contrast, when training the algorithms with the unselected pain thresholds, to which belonged the thermal pain thresholds and the effects of sensitization, they could not identify the subjects’ sex better than by guessing. Classification performance was then equivalent to that obtained when permuted data, i.e., nonsense information, was used for training (Fig. 4). This underscores that the informative pain threshold information in the sex context had been indeed identified during feature selection (Fig. 4).

#### Possible molecular-pharmacological implications

**Figure 5:**
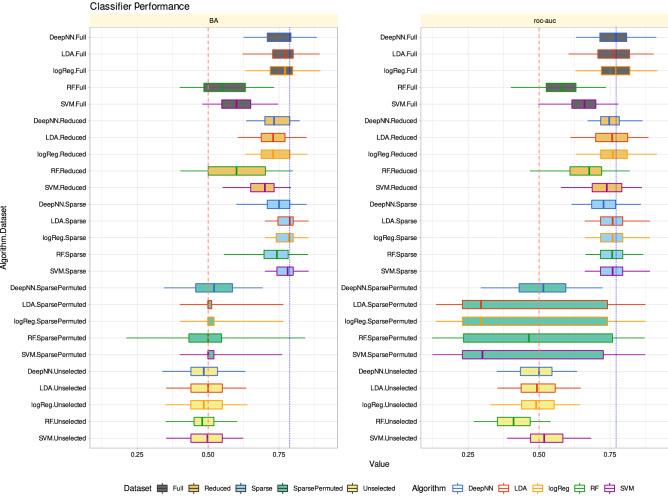
Performance of different classifier types (linear support vector machine, SVM, random forests, logistic regression, linear discriminant analysis, LDA) in assigning sex in the validation data set. The classifiers were trained in a 100-fold nested cross validation setting with subsets randomly drawn from the 80% training data set separated at the beginning of the analyses. Training was performed with all variables (d = 11 pain-threshold related variables) as “full” feature set, d = 4 variables that had resulted from the feature selection steps shown in Fig. 4 as “reduced” feature set (darker blue columns Fig. 4A), or d = 1 variable that had resulted from further narrowing the feature set to the “sparse” feature set (darkest blue column Fig. 4A). In addition, the sex assignment task was repeated using permuted data for algorithm training or using the unselected features, i.e., the pain-threshold information that was not found to be informative for sex inference (light blue columns in Fig. 4A). The boxes show the 25th, 50th and 75th percentiles balanced accuracy (BA) and roc-auc for the classification performance in the 20% validation data separated before feature selection and classifier training from the whole data set and not used for feature selection and algorithm training. Whiskers span the 95% confidence interval from the 2.5th to the 97.5th percentiles. The vertical dashed red lines mark the 50% guessing level that must not be touched by the confidence interval of the classification performance measures if the algorithm can be considered successfully trained. The vertical dotted blue lines mark the respective best median classification performance observed across all data set variants and algorithms. The figure has been created using the software package R (version 4.2.2 for Linux; https://CRAN.R-project.org/^6^) and the R libraries "ggplot2" (https://cran.r-project.org/package=ggplot2^7^) and "ComplexHeatmap" (https://bioconductor.org/packages/release/bioc/html/ComplexHeatmap.html^58^.

Evidence identified by literature search for nociceptors involved in the perception of pain elicited by mechanical but not thermal noxious stimuli, nor by the hypersensitization induced by capsaicin and menthol, pointed to d = 13 molecular targets (Table 2). Information retrieval from the DrugBank database provided a 79 × 13 matrix of drugs versus nociceptive targets (Fig. 5). Most of the 79 drugs were calcium channel blockers targeting cardiovascular diseases and might be of interest for drug repurposing to target pain. Of the in total 14,594 unique entries in the DrugBank database, 229 drugs were annotated with a therapeutic indication that contained the substrings “pain” or “nocicep” or “analge”. Of those, 10 were among the 79 drugs in above matrix, namely tramadol, enflurane, methadone, chlorzoxazone, meperidine, levomenthol, ethanol, orphenadrine, cns-5161, ketobemidone. Most are well-known substances. Of the lesser-known ones, orphenadrine is mentioned in the DrugBank database as a muscarinic antagonist used to treat drug-induced parkinsonism and to relieve pain from muscle spasm, and CNS-5161 is a *N*-methyl-d-aspartate ion channel antagonist.

**Table 2:** Molecular sensors of mechanical pain for which no positive evidence was found that they also transmit thermal nociception, or for which negative evidence has been found with respect to an involvement in thermal nociception.

Gene symbol	Gene name	NCBI number	Gene name via NCBI	UNIPROT Id	Reference
*ASIC1*	Acid sensing ion channel subunit 1	41	*ASIC1*	A8K1U5	^48^
*ASIC2*	Acid sensing ion channel subunit 2	40	*ASIC2*	Q16515	^48^
*ASIC3*	Acid sensing ion channel subunit 3	9311	*ASIC3*	A0A090N7X8	^48^
*CACNA1C*	Calcium voltage-gated channel subunit alpha1 C	775	*CACNA1C*	Q13936	^49^
*FXYD2*	FXYD domain containing ion transport regulator 2	486	*FXYD2*	P54710	^50^
*GRIN1*	Glutamate ionotropic receptor NMDA type subunit 1	2902	*GRIN1*	Q05586	^51^
*HCN2*	Hyperpolarization activated cyclic nucleotide gated potassium and sodium channel 2	610	*HCN2*	Q9UL51	^52^
*KCNJ8*	Potassium inwardly rectifying channel subfamily J member 8	3764	*KCNJ8*	A0A024RAV6	^53^
*P2RY6*	Pyrimidinergic receptor P2RY6	5031	*P2RY6*	A0A024R5I9	^54^
*PIEZO1*	Piezo type mechanosensitive ion channel component 1	9780	*PIEZO1*	Q92508	^55^
*PIEZO2*	Piezo type mechanosensitive ion channel component 2	63,895	*PIEZO2*	Q9H5I5	^55^
*SCNN1A*	Sodium channel epithelial 1 subunit alpha	6337	*SCNN1A*	P37088	^56^
*SLO1*	Potassium calcium-activated channel subfamily M alpha 1	3778	*KCNMA1*	Q12791	^57^

The gene symbols used in the original publication are given along with the actual symbols retrieved via the NCBI number using the R library "org.Hs.eg.db" https://bioconductor.org/packages/release/data/annotation/html/org.Hs.eg.db.html^47^).

## Discussion

**Figure 6:**
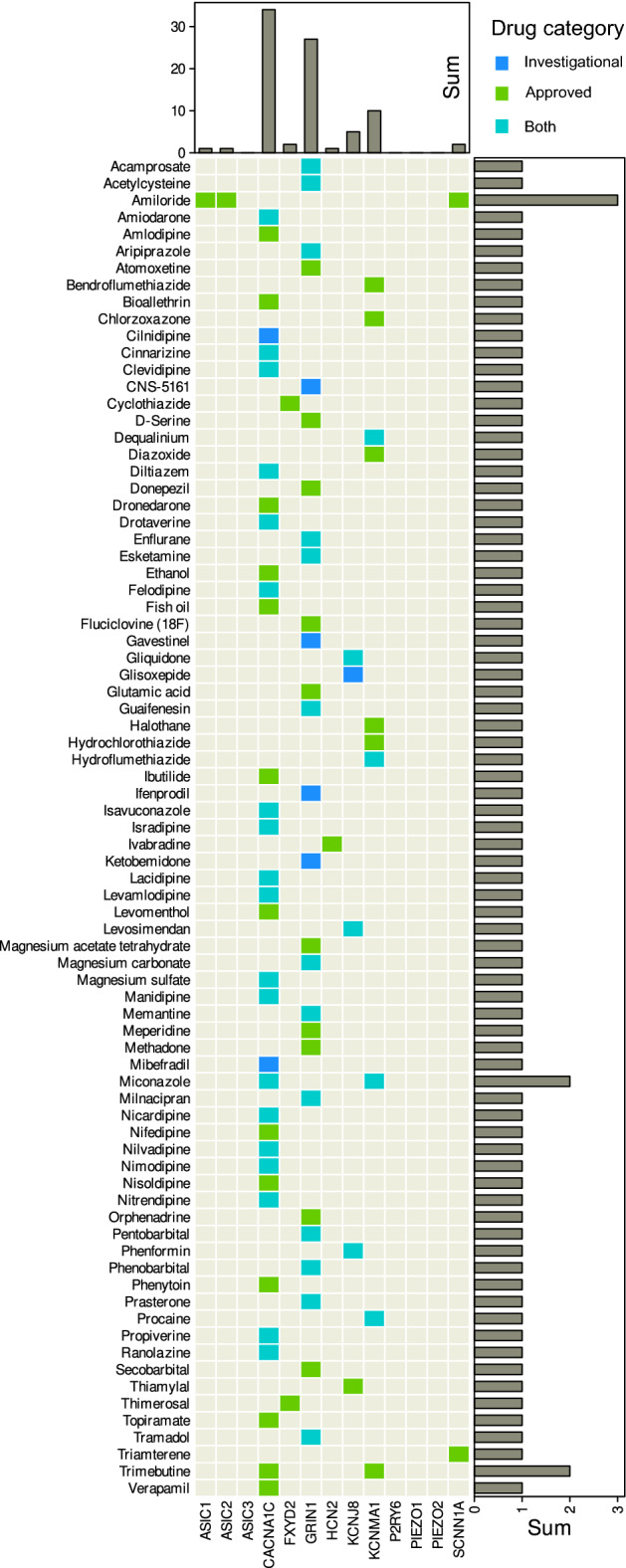
Matrix heat plot of 79 × 13 drugs versus targets among nociceptors involved in pain triggered by mechanical stimuli (Table 2). Shown are approved and/or investigational drugs that were queried from the DrugBank database along with their targets (https://go.drugbank.com^44^). Marginal statistics (sums) are shown as bar graphs indicating row and column sums of interactions per drug and target, respectively. The figure was created using the R software package (version 4.2.2 for Linux; https://CRAN.R-project.org/ (R Development Core Team, 2008)) and the R library "ComplexHeatmap" (https://bioconductor.org/packages/release/bioc/html/ComplexHeatmap.html^58^).

The application of machine learning principles^59^ allowed the inversion of the association of sex and pain sensitivity. A median balanced accuracy of 74–79% can be considered as good according to the usual criteria for machine learning classifiers. This strongly indicates that a person’s sex is highly relevant in pain research and probably in pain treatment. The analyses showed that sex segregation is not evenly distributed across the pain thresholds to different noxious stimuli. Positive evidence was provided that sex can be inferred from mechanical thresholds and negative evidence that it cannot be inferred from thermal thresholds or from the effects of capsaicin/menthol induced hypersensitization. The "not" logic, based on consideration of the unselected features^43^, is likely to hold as well to other machine learning algorithms than the presently used selection, which already covers various architectures up to "deep learning". None of the algorithms showed any tendency to leave the guessing range when trained with the unselected features, i.e., thermal pain thresholds or sensitization effects (Fig. 6).

### Implications for pain precision medicine

The logic of "mechanical but not thermal" can be directly translated to the molecular level by focusing on pathways. Three main types of nociceptors are expressed in the skin: mechanosensitive and mechanothermal nociceptors, which signal mainly through myelinated A_δ_-nerve fibers, and polymodal nociceptors, which are connected to unmyelinated C-nerve fibers^60^. Mechanical and thermal stimuli elicit pain through different pathways; for example, neurons activated by the peripheral application of heat differ from those activated by noxious pressure^61^. Mechanoreceptors and mechano-transducers include ion channels, specialized cytoskeletal proteins, cell junction molecules, and G protein-coupled receptors. There is growing evidence that mechanical stimuli play a role in pathological pain conditions^62–64^.

Ion channels that have been shown to be involved in the perception of mechanical stimuli include the so-called piezo-receptors, PIEZO1 and PIEZO2, which are currently the largest known ion channels with three identical subunits of approximately 2500 amino acids each and are highly conserved in vertebrates^65^. These ion channels control the uptake of cationic ions, particularly Ca^2+^, in response to a stimulus, allowing them to mediate action potentials and intracellular signal transduction through second messenger signaling^66^. Ca^2+^ entry mediated by mechanosensitive ion channels is also tightly regulated by a variety of key proteins distributed in the cell membrane or endoplasmic reticulum. Recent studies have shown that mechanosensitive ion channels can even physically interact with Ca^2+^-regulatory proteins, and these interactions have far-reaching implications for physiology and pathophysiology^67^.

The frequency of action potentials in nociceptors is a major determinant of pain intensity. Modulators include HCN ion channels, which generate an inward current following membrane hyperpolarization^68^. The human HCN2 channel is activated by hyperpolarization that exhibits weak selectivity for potassium versus sodium ions^69^. It contributes to native pacemaker currents in the heart and neurons. These channels have recently been linked to chronic pain. Blocking the *HCN2* gene has been associated with the disappearance of neuropathic pain episodes^70^. In addition, HCN2 has been shown to interact with HCN1^71^ and HCN4^71^ by co-assembling homologous subunits to heteromeric complexes.

One possible mechanism for the sex-specificity of mechanical nociceptors could be an allosteric interaction with estrogens. That is, while receptors are physiologically stimulated by endogenous orthosteric agonists, few naturally occurring allosteric modulators have been described^72^. Steroid hormones such as progesterone and estradiol exert allosteric effects on cannabinoid^73^, oxytocin^74^, and glycoprotein hormone receptors^75^, respectively, implying that receptors can be indirectly modulated by changing the concentration of the endogenous allosteric modulator^76^. Recently, neurosteroids and neuroactive steroids were shown to act as allosteric modulators of muscarinic receptors, which are a subfamily of G-protein receptors^77,78^ and GABA receptors, a class of receptors that respond to the neurotransmitter gamma-aminobutyric acid (GABA), the major inhibitory compound in the mature vertebrate central nervous system^79^. However, the relevance of these allosteric mechanisms to sex-specific mechanical pain remains to be demonstrated, as corresponding evidence for mechanical nociceptors is still lacking in the literature.

Pharmacological application of allosteric modulation is conceivable with emerging approaches to drug design based on secondary binding site effects, where small molecule drugs are designed to bind into secondary binding sites on the targeted biomolecules^80^. According to the present line of reasoning, drugs addressing the molecular targets of mechanical nociceptive stimuli might act better on pain in women than in men and thus be suitable for sex-specific analgesic treatment. For the 10 drugs annotated with effects on pain, evidence for a better effect in women is sparse^81^. Nonetheless, the present explorative analysis illustrates the possibility that the data analysis approach presented in this report could be used for sex-specific precision medicine in pain. Certain analgesics could be preferred or future approaches to drug repurposing could be guided. Of course, this would also require the identification of clinical pain settings in which mechanical stimuli play a main role. To date, sex specific analgesia has been rarely implemented clinically. However, proof-of-concept is provided through the observation that κ-opioid agonists (pentazocine) are more efficient in women with the "red hair—white skin" phenotype based on variants of the melanocortin-1 receptor gene (*MC1R*)^82^.

While it seems accepted that females have lower pain thresholds for various forms of noxious stimuli^83^, the mechanistic basis of this sexual dimorphism in nociception is far from being fully understood. Transcriptome analyses of the whole dorsal root ganglion of female rats have revealed lower cutaneous mechanical nociceptive thresholds^84^. Several studies have shown that in antinociceptive procedures using thermal, chemical and electrical stimuli, opioids are generally more potent in male than in female rodents, and vice versa^85^. It has also been shown that temporal summation of heat pain is greater in females than in males, suggesting that central processing of nociceptive input may be upregulated in females^86^. Several further studies have investigated temporal summation to noxious mechanical stimulation and examined sex differences in temporal summation of mechanically evoked pain^87^. Their results indicate that temporal summation of mechanically evoked pain is higher in females than in males, is stimulus frequency dependent, and is centrally mediated^86^.

The absence of ovarian hormones caused an increase in mean arterial pressure and a decrease in paw nociception in female rodents. Ovarian hormones play an important role in the mechanical threshold. The ovaries secrete two important hormones, estrogen and progesterone, which play a role in pain modulation^88^. Furthermore, many chronic pain conditions show sex differences in their epidemiology^89^. This may be due to sex-specific differential expression of genes involved in nociceptive pathways, including immune cells, sensory neurons, and the neuroendocrine system^90^. Elucidating how different cell types detect and respond to noxious stimuli may lead to new therapeutic insights and a better understanding of the basic mechanisms of pain plasticity.

### Implications for pain data analysis

Seeking structure in data sets can be complex and the standard approach of perform a principal component analysis (PCA) to project data followed by group segregation statistics may not suffice always. In the present data set, if any structure and clusters could be detected in the data with unsupervised analyses (for a complete description of methods and results of unsupervised analyses, see Supplementary information), then after t-SNE (t-distributed stochastic neighborhood embedding) projection (Supplementary Fig. 5). Supervised approaches to structures that reflected the sex structure in the present data provided more convincing results. However, it was not possible to determine with certainty in advance which method was successful in the present data set. This emphasizes that a complete analysis of pain-related data structures should include several methods.

Second, by using a machine-learning approach, the research question of sex differences in pain could be reversed by addressing the question whether pain can be used to infer sex. This allowed address negative evidence with the thermal pain thresholds directly. That is, the absence of positive evidence as provided in statistically non-significant sex differences in thermal pain thresholds could be extended to a presence of negative evidence by showing that sex inference from thermal thresholds was not better than guessing, similar to training the algorithms with “nonsense” information created by permutation of the variables. This emphasizes the need for negative evidence, regarded as evidence for non-involvement and not only as absent evidence for involvement, which is rarely provided in the scientific literature. For the present molecular targets (Table 2), explicit evidence for non-involvement in thermal pain was found only for HCN2^91^.

Finally, it should be noted that the present analyses cannot cover the full possible spectrum of AI and machine learning in pain research. While several different methods of data analysis were implemented, it is not possible to cover all (for an overview, see^58^). Similarly, the deep learning neural network^32,33^ was trained only in one of the discriminative forms (for an overview, see e.g.^59^) while further architectures were not tested. However, without including other architectures, the utility of deep learning for datasets such as the present one cannot be conclusively judged.

## Conclusions

Formulating the question of sex differences in pain as a machine learning problem allowed for an unbiased analysis that concluded that pain thresholds differ relevantly, to the extent that algorithms can be trained with pain threshold information from men and women that they are able to correctly infer sex in new subjects by up to 79% balanced accuracy. This underscores that sex cannot be ignored in pain research and probably in analgesic therapy. By addressing a subsequent hypothesis that included a "NOT" logic, machine learning indicated that sex-relevant information resides in the pain thresholds for mechanical but not thermal stimuli of responses to local hypersensitization. The hypothesis thus formulated could be translated into a molecular hypothesis about nociceptive targets. The present analytic approach highlights that a diverse composition of data science methods including machine learning algorithms can help decipher deep data structures relevant to biomedical and clinical professionals.

## Supplementary Information

Supplementary Information.

## Data Availability

Data are available from the first author on request.

## References

[1] Fillingim RB, King CD, Ribeiro-Dasilva MC, Rahim-Williams B, Riley JL (2009). Sex, gender, and pain: A review of recent clinical and experimental findings. J. Pain.

[2] Derbyshire SW (2008). Gender, pain, and the brain. Pain Clin. Updates.

[3] Unruh AM (1996). Gender variations in clinical pain experience. Pain.

[4] Petrovich DV (1959). The pain apperception test: An application to sex differences. J. Clin. Psychol..

[5] Kovalchik, S. RISmed: Download Content from NCBI Databases. R package version 2.3.0, https://CRAN.R-project.org/package=RISmed (2021).

[6] R Development Core Team (2008). R: A Language and Environment for Statistical Computing.

[7] Wickham H (2009). ggplot2: Elegant Graphics for Data Analysis.

[8] Samuel AL (1959). Some studies in machine learning using the game of checkers. IBM J. Res. Dev..

[9] Doehring A (2011). Effect sizes in experimental pain produced by gender, genetic variants and sensitization procedures. PLoS One.

[10] Lötsch J, Ultsch A, Mayer B, Kringel D (2022). Artificial intelligence and machine learning in pain research: A data scientometric analysis. Pain Rep..

[11] Petersen KL, Rowbotham MC (1999). A new human experimental pain model: The heat/capsaicin sensitization model. NeuroReport.

[12] Hatem S, Attal N, Willer JC, Bouhassira D (2006). Psychophysical study of the effects of topical application of menthol in healthy volunteers. Pain.

[13] Lotsch J, Ultsch A (2017). Machine learning in pain research. Pain.

[14] Guyon I (2003). An introduction to variable and feature selection. J. Mach. Learn. Res..

[15] Ihaka R, Gentleman R (1996). R: A language for data analysis and graphics. J. Comput. Graph. Stat..

[16] Van Rossum G, Drake FL (1995). Python Tutorial.

[17] Pearson KLIII (1901). On lines and planes of closest fit to systems of points in space. Lond. Edinb. Dublin Philos. Mag. J. Sci..

[18] Hyvärinen A, Oja E (2000). Independent component analysis: Algorithms and applications. Neural Netw..

[19] Shepard RN (1962). The analysis of proximities: Multidimensional scaling with an unknown distance function. II. Psychometrika.

[20] Shepard RN (1962). The analysis of proximities: Multidimensional scaling with an unknown distance function. I. Psychometrika.

[21] Tenenbaum JB, de Silva V, Langford JC (2000). A global geometric framework for nonlinear dimensionality reduction. Science.

[22] Van der Maaten L, Hinton G (2008). Visualizing data using t-SNE. J. Mach. Learn. Res..

[23] MacQueen, J. In *Proceedings of the Fifth Berkeley Symposium on Mathematical Statistics and Probability, Volume 1: Statistics.* 281–297 (University of California Press).

[24] Kaufman L, Rousseeuw PJ (1990). Partitioning around medoids (program PAM). Finding Groups Data.

[25] Ward JH (1963). Hierarchical grouping to optimize an objective function. J. Am. Stat. Assoc..

[26] Cortes C, Vapnik V (1995). Support-vector networks. Mach. Learn..

[27] Ho, T. K. In *Proceedings of the Third International Conference on Document Analysis and Recognition (Volume 1)—Volume 1* 278 (IEEE Computer Society, 1995).

[28] Breiman L (2001). Random forests. Mach. Learn..

[29] Cramer JS (2002). The Origins of Logistic Regression.

[30] Fisher RA (1936). The use of multiple measurements in taxonomic problems. Ann. Eugen..

[31] Deng L, Yu D (2014). Deep learning: Methods and applications. Found. Trends Signal Process..

[32] McCulloch WS, Pitts W (1943). A logical calculus of the ideas immanent in nervous activity. Bull. Math. Biophys..

[33] Hinton GE, Osindero S, Teh Y-W (2006). A fast learning algorithm for deep belief nets. Neural Comput..

[34] Lötsch J, Malkusch S, Ultsch A (2021). Optimal distribution-preserving downsampling of large biomedical data sets (opdisDownsampling). PLoS One.

[35] Pedregosa F (2011). Scikit-learn: Machine learning in Python. J. Mach. Learn. Res..

[36] Ultsch A, Lötsch J (2015). Computed ABC analysis for rational selection of most informative variables in multivariate data. PLoS One.

[37] Juran JM (1975). The non-Pareto principle; Mea culpa. Qual. Progress.

[38] Lötsch J, Ultsch A (2023). Recursive computed ABC (cABC) analysis as a precise method for reducing machine learning based feature sets to their minimum informative size. Sci Rep..

[39] Abadi, M. n. *et al.* TensorFlow: Large-Scale Machine Learning on Heterogeneous Systems. CoRR. vol. abs/1603.04467. http://arxiv.org/abs/1603.04467 (2016).

[40] Waskom ML (2021). seaborn: Statistical data visualization. J. Open Source Softw..

[41] Brodersen, K. H., Ong, C. S., Stephan, K. E. & Buhmann, J. M. In *Pattern Recognition (ICPR), 2010 20th International Conference on.* 3121–3124.

[42] Peterson W, Birdsall T, Fox W (1954). The theory of signal detectability. Trans. IRE Prof. Group Inf. Theory.

[43] Lötsch J, Ultsch A (2022). Enhancing explainable machine learning by reconsidering initially unselected items in feature selection for classification. BioMedInformatics.

[44] Wishart DS (2018). DrugBank 5.0: A major update to the DrugBank database for 2018. Nucleic Acids Res..

[45] Ali, M. & Ezzat, A. dbparser: DrugBank Database XML Parser. R package version 1.2.0. https://cran.r-project.org/package=dbparser (2020).

[46] UniProt: The universal protein knowledgebase in 2021. *Nucleic Acids Res. ***49**, D480–D489. 10.1093/nar/gkaa1100 (2021). 10.1093/nar/gkaa1100 PMC7778908 33237286

[47] Carlson, M. org.Hs.eg.db: Genome wide annotation for Human. R package version 3.16.0. https://bioconductor.org/packages/release/data/annotation/html/org.Hs.eg.db.html (2020).

[48] Ruan N (2021). Acid-sensing ion channels and mechanosensation. Int. J. Mol. Sci..

[49] Efremov AK (2022). Application of piconewton forces to individual filopodia reveals mechanosensory role of L-type Ca2+ channels. Biomaterials.

[50] Ventéo S (2016). Fxyd2 regulates Aδ- and C-fiber mechanosensitivity and is required for the maintenance of neuropathic pain. Sci. Rep..

[51] Paoletti P, Ascher P (1994). Mechanosensitivity of NMDA receptors in cultured mouse central neurons. Neuron.

[52] Emery EC, Young GT, McNaughton PA (2012). HCN2 ion channels: An emerging role as the pacemakers of pain. Trends Pharmacol. Sci..

[53] Al-Shammari H (2020). Expression and function of mechanosensitive ion channels in human valve interstitial cells. PLoS One.

[54] Kauffenstein G (2016). Central role of P2Y6 UDP receptor in arteriolar myogenic tone. Arterioscler. Thromb. Vasc. Biol..

[55] Coste B (2012). Piezo proteins are pore-forming subunits of mechanically activated channels. Nature.

[56] Rossier BC (1998). Mechanosensitivity of the epithelial sodium channel (ENaC): Controversy or pseudocontroversy?. J. Gen. Physiol..

[57] Geng Y, Magleby KL (2014). Single-channel kinetics of BK (Slo1) channels. Front. Physiol..

[58] Gu Z, Eils R, Schlesner M (2016). Complex heatmaps reveal patterns and correlations in multidimensional genomic data. Bioinformatics.

[59] Murphy KP (2012). Machine Learning: A Probabilistic Perspective.

[60] Eilers H, Schumacher MA, Kamkin A, Kiseleva I (2005). Mechanosensitivity in Cells and Tissues.

[61] Lariviere WR (2002). Heritability of nociception. III. Genetic relationships among commonly used assays of nociception and hypersensitivity. Pain.

[62] Lolignier S, Eijkelkamp N, Wood JN (2015). Mechanical allodynia. Pflugers Arch..

[63] Kuner R (2010). Central mechanisms of pathological pain. Nat. Med..

[64] Coderre TJ, Katz J, Vaccarino AL, Melzack R (1993). Contribution of central neuroplasticity to pathological pain: Review of clinical and experimental evidence. Pain.

[65] Zarychanski R (2012). Mutations in the mechanotransduction protein PIEZO1 are associated with hereditary xerocytosis. Blood.

[66] Chubinskiy-Nadezhdin VI (2017). Local calcium signalling is mediated by mechanosensitive ion channels in mesenchymal stem cells. Biochem. Biophys. Res. Commun..

[67] Wang Y, Shi J, Tong X (2021). Cross-talk between mechanosensitive ion channels and calcium regulatory proteins in cardiovascular health and disease. Int. J. Mol. Sci..

[68] Emery EC, Young GT, Berrocoso EM, Chen L, McNaughton PA (2011). HCN2 ion channels play a central role in inflammatory and neuropathic pain. Science.

[69] Vaccari T (1999). The human gene coding for HCN2, a pacemaker channel of the heart. Biochim. Biophys. Acta.

[70] Lainez S, Tsantoulas C, Biel M, McNaughton PA (2019). HCN3 ion channels: Roles in sensory neuronal excitability and pain. J. Physiol..

[71] Proenza C (2002). Different roles for the cyclic nucleotide binding domain and amino terminus in assembly and expression of hyperpolarization-activated, cyclic nucleotide-gated channels. J. Biol. Chem..

[72] Melancon BJ (2012). Allosteric modulation of seven transmembrane spanning receptors: Theory, practice, and opportunities for central nervous system drug discovery. J. Med. Chem..

[73] Morales P, Goya P, Jagerovic N, Hernandez-Folgado L (2016). Allosteric modulators of the CB(1) cannabinoid receptor: A structural update review. Cannabis Cannabinoid. Res..

[74] Grazzini E, Guillon G, Mouillac B, Zingg HH (1998). Inhibition of oxytocin receptor function by direct binding of progesterone. Nature.

[75] Rossi M (2009). Presence of a putative steroidal allosteric site on glycoprotein hormone receptors. Eur. J. Pharmacol..

[76] Fasciani I (2020). Allosteric modulators of G protein-coupled dopamine and serotonin receptors: A new class of atypical antipsychotics. Pharmaceuticals (Basel).

[77] Dolejší E (2021). Neurosteroids and steroid hormones are allosteric modulators of muscarinic receptors. Neuropharmacology.

[78] Szczurowska E, Szánti-Pintér E, Randáková A, Jakubík J, Kudova E (2022). Allosteric modulation of muscarinic receptors by cholesterol, neurosteroids and neuroactive steroids. Int. J. Mol. Sci..

[79] Martinez Botella G (2015). Neuroactive steroids. 1. Positive allosteric modulators of the (γ-aminobutyric acid)A receptor: Structure–activity relationships of heterocyclic substitution at C-21. J. Med. Chem..

[80] Abdel-Magid AF (2015). Allosteric modulators: An emerging concept in drug discovery. ACS Med. Chem. Lett..

[81] Rauck RL (2006). A randomized, double-blind, placebo-controlled study of intrathecal ziconotide in adults with severe chronic pain. J. Pain Symptom Manage..

[82] Mogil JS (2003). The melanocortin-1 receptor gene mediates female-specific mechanisms of analgesia in mice and humans. Proc. Natl. Acad. Sci. USA.

[83] Bartley EJ, Fillingim RB (2013). Sex differences in pain: A brief review of clinical and experimental findings. Br. J. Anaesth..

[84] Hendrich J (2012). In vivo and in vitro comparison of female and male nociceptors. J. Pain.

[85] Barrett AC, Smith ES, Picker MJ (2002). Sex-related differences in mechanical nociception and antinociception produced by mu- and kappa-opioid receptor agonists in rats. Eur. J. Pharmacol..

[86] Sarlani E, Greenspan JD (2002). Gender differences in temporal summation of mechanically evoked pain. Pain.

[87] Riley JL, Robinson ME, Wise EA, Myers CD, Fillingim RB (1998). Sex differences in the perception of noxious experimental stimuli: A meta-analysis. Pain.

[88] Chen Q, Zhang W, Sadana N, Chen X (2021). Estrogen receptors in pain modulation: Cellular signaling. Biol. Sex Differ..

[89] Mecklenburg J (2020). Transcriptomic sex differences in sensory neuronal populations of mice. Sci. Rep..

[90] Paige C (2020). Neuroendocrine mechanisms governing sex differences in hyperalgesic priming involve prolactin receptor sensory neuron signaling. J. Neurosci..

[91] Schnorr S (2014). HCN2 channels account for mechanical (but not heat) hyperalgesia during long-standing inflammation. Pain.

