# Supplementary material for: Machine learning analysis predicts a person’s sex based on mechanical but not thermal pain thresholds
Source: Sci Rep. 2023 May 5;13:7332. doi: 10.1038/s41598-023-33337-2 (PMC10163041; doi:10.1038/s41598-023-33337-2)
Supplement: Supplementary file 1 — Supplementary Information. [file 41598_2023_33337_MOESM1_ESM.pdf]

Supplemental information: Data preprocessing and description of methods and results of unsupervised data analyses

Data preprocessing

Examination of the distribution of the variables included evaluation of possible transformations along Tukey's ladder of powers [1,2], supported by visualizing the data using quantile-quantile plots and assessing the normal distribution using D'Agostino and Pearson tests [3,4] implemented in the "SciPy" Python package (https://scipy.org [5]). This suggested logarithmic transformation, which is in line with the > 150 years old law of Weber and Fechner [6]. Statistical group comparisons with Wilcoxon-Mann Whitney U tests [7,8] revealed sex differences for some thresholds, of which only pressure pain thresholds passed Bonferroni's α correction [9] (Figure 2 in the main report).

Inspection of the data distribution identified n = 30 and n = 19 censored values in the pain thresholds for cold stimuli recorded without and after sensitization with menthol solution, respectively, at the cut-off of thermal stimulation at a temperature of 0°C. In addition, n = 38 and n = 21 censored values were found in the pain thresholds for punctate stimuli recorded without and after sensitization with capsaicin cream, respectively, at the thickest von Frey hair of 300 g. These values were replaced using censored likelihood multiple imputation [10] implemented in the R library "lodi" (https://cran.r-project.org/package=lodi [11]). Specifically, censored values were first replaced with "NA" (no value) and then imputed, using variables that were correlated with a Spearman's $\rho > 0.4$ [12] with the variable being imputed (Supplemental Figure 1), i.e., heat thresholds for cold thresholds and heat and blunt pressure thresholds for punctate pressure thresholds, omitting correlated variables from the imputation process that also contained censored values. The relatively low correlation of $\rho > 0.4$ was used as a criterion because the overall intercorrelation of pain thresholds was not very strong and the use of other censored variables for imputation was avoided. The results of the imputation are shown in the Supplemental Figure 1.

Unsupervised analysis of structures in the pain threshold data

Methods

Unsupervised analyses

The first line of structure detection was based on unsupervised analysis of traces of prior classes, i.e., sex, in the acquired pattern of pain thresholds to noxious stimuli across the study cohort.

Univariate structure detection

Unsupervised structure detection in the pain-related data was performed firstly by univariate assessment of the modal distribution of pain thresholds after transformation and imputation during data preprocessing. Therefore, Gaussian mixture modeling was applied to the pain threshold variables, testing M = [1,...,8] modes and using goodness of fit as a selection criterion for the number of modes, which was evaluated by performing a likelihood ratio test of the distribution according to the Gaussian fits compared with the distributions of the original data. The GMM fitting was performed using an expectation maximization (EM) algorithm, which was imported from the R library "mixtools" (https://cran.r-project.org/package=mixtools [13]) into our R package "opGMMassessment" (https://cran.r-project.org/package=opGMMassessment [14], which provides an automated tool for detecting GMM-based structures in (biomedical) datasets.

The assignment of subjects to the identified subgroups was determined using Bayesian Theorem [15], which provides the decision limits for assigning a single observation to mode M_i based on the calculation of posterior probabilities. The correspondence of the group assignment based on the Gaussian modes in the relevant PCs with the *a priori* subgroup distribution was statistically evaluated using χ^2 tests [16].

Multivariate structure detection

Unsupervised structure detection in the pain-related data was performed secondly by multivariate analyses consisting of projecting the data from high-dimensional space onto a two-dimensional $\mathbb{R}^2$ plane., followed by cluster analysis and assessment of the constancy between the clusters and the prior classes. The number of clusters was defined as k = 2, given by the number of prior classes, i.e., sex.

Projection methods included principal component analysis [17] as the most used method for clinical and other biomedical data. To address possible structures based on independent components, non-linear relationships between variables or dependent more on neighborhood than on variance in the data, further projection methods were applied including independent component analysis (ICA) [18], multidimensional scaling (MDS) [19,20], isomap [21] and t-distributed stochastic neighborhood embedding (t-SNE) [22]. The data were projected after z-standardization using the R packages "FactoMineR" (https://cran.r-project.org/package=FactoMineR [23]), "FastICA" (https://cran.r-project.org/package=fastICA [24]), "Rtsne" (https://cran.r-project.org/package=Rtsne [25]), “MASS” (https://cran.r-project.org/package=MASS [26]) and "RDRToolbox" (https://bioconductor.org/packages/RDRToolbox/ [27]) with their default hyperparameter settings.

Clustering on projected data was performed using partition-based and agglomerative algorithms, including k-means clustering [28] and partitioning around medoids (PAM) [29] as well as hierarchical clustering with Ward's [30], average, single, median and complete linkage in analogy to or extending the choice made in [31]. The Euclidean distance was used. Clustering was done using the R packages “stats” and “cluster” from the R base environment (https://cran.r-project.org/package=cluster [32]). Calculations were performed using the default hyperparameter settings of the methods specified in the libraries, except for k-means where the number of restarts of the searches was set at nstart = 10. Contingency between cluster membership and prior class membership of cases was assessed using the

χ^2 statistics [16]. This was repeated in a 100-fold bootstrap resampling test to obtain confidence intervals of the p-values.

Results

Prior-classes separation at the data projection planes obtained in unsupervised analyses

Univariate class structure

Automated fitting of Gaussian mixture models identified M = 1 to 6 modes in pain thresholds to various stimuli, transformed and imputed for censored data, including changes in pain thresholds after sensitization with capsaicin or menthol (Supplemental Figure 2). Assignment to the obtained Gaussian modes that also resulted in a statistically significant sex separation was observed for the thresholds to blunt pressure and to electrical stimuli (χ^2-tests: $p < 0.05$; Supplemental Figure 2); the one for pressure pain with $p = 0.00065$ also passed α correction.

Multivariate class structure

The data projection obtained with the five different methods showed a scattered image of the two prior classes with no clear class segregation (Supplemental Figure 3). Furthermore, clustering the data at the $\mathbb{R}^2$ plane provided heterogeneous results without the clusters appearing to capture the prior classes (Supplemental Figure 4). Hierarchical clustering using single linkage produced obviously erroneous results, as one "cluster" contained only a single case, while all other cases were assigned to the second cluster (Supplemental Figure 4 fourths line of panels from top). The heterogeneous overall picture about contingency between clusters and prior classes was reflected in the statistical significances. When using the full data set once in a cross tabulation of cluster versus prior class memberships, nine combinations of clustering algorithms and projection methods provided significant results, with the lowest p values obtained with were Ward’s linkage on ICA projected data (p = 0.005707688), average linkage on the tSNE projection (p = 0.012931459) and kmeans on the tSNE projection (p = 0.017159885), and the highest still significant finding for complete linkage applied on

PCA projected data (p = 0.039483512). However, in evaluating the contingency tables in 100-fold bootstrap cross-validation tests, two main observations emerged from the overall picture of statistical results (Supplemental Figure 5), i.e., t-SNE projection was on the top of the ranking for p-values, with median p-values most often below the value of 0.05, while hierarchical clustering with simple linkage provided the worst separation of priority classes in the clusters, as measured by the significance of the cross-tabulation tests. The latter is consistent with the picture that emerged when the prior classes were plotted on a projection plane consisting of Voronoi cells colored for cluster membership of each data point in a cell (Supplemental Figure 4).

Summary of findings with unsupervised analyses

Unsupervised analysis using five different common data projection methods and seven different common clustering algorithms ended up with a heterogeneous picture about the data structure and its correspondence to the prior classes (Supplemental Figure 3 and Supplemental Figure 4). For example, if only the usual standard PCA had been used, then only hierarchical clustering with Ward's or complete linkage would have led to the conclusion that pain thresholds supported the segregation of subjects' sensitivity by sex. However, 100-fold bootstrap resampling from the clusters already lowered the statistical significance of the agreement of the cluster membership with the subjects' sex, thus weakening the support for a cluster structure that coincided with the sex segregation. In the 100-fold bootstrap resampling runs, t-SNE was found to have provided the best data projection on which clustering could be based when agreement with the sex segregation was the criterion, except for hierarchical clustering using single linkage that always produced poor results. However, t-SNE is more difficult to set up than the classic PCA as it requires the setting of hyperparameters such as a so-called perplexity size [33], and it my occasionally produce spurious result [34].

References

1 Tukey, J. W. & others. *Exploratory data analysis*. Vol. 2 (Reading, Mass., 1977).

2 Box, G. E. & Cox, D. R. An analysis of transformations. *Journal of the Royal Statistical Society. Series B (Methodological)*, 211-252 (1964).

3 D'Agostino, R. & Pearson, E. S. Tests for Departure from Normality. Empirical Results for the Distributions of b₂ and √ b₁. *Biometrika* **60**, 613-622, doi:10.2307/2335012 (1973).

4 D'Agostino, R. B. An omnibus test of normality for moderate and large size samples. *Biometrika* **58**, 341-348, doi:10.1093/biomet/58.2.341 (1971).

5 Virtanen, P. *et al.* SciPy 1.0: fundamental algorithms for scientific computing in Python. *Nat Methods* **17**, 261-272, doi:10.1038/s41592-019-0686-2 (2020).

6 Fechner, G. T. *Elemente der Psychophysik*. (Breitkopf and Härtel, 1860).

7 Wilcoxon, F. Individual comparisons by ranking methods. *Biometrics* **1**, 80-83 (1945).

8 Mann, H. B. & Whitney, D. R. On a test of whether one of two random variables is stochastically larger than the other. *Annals of Mathematical Statistics* **18**, 50-60 (1947).

9 Bonferroni, C. E. Teoria statistica delle classi e calcolo delle probabilita. *Pubblicazioni del R Istituto Superiore di Scienze Economiche e Commerciali di Firenze* **8**, 3-62, doi:citeulike-article-id:1778138 (1936).

10 Boss, J. *et al.* Estimating Outcome-Exposure Associations when Exposure Biomarker Detection Limits vary Across Batches. *Epidemiology* **30** (2019).

11 Boss, J. & Rix, A. (2020).

12 Spearman, C. The proof and measurement of association between two things. *Am J Psychol* **15**, 72–101 (1904).

13 Benaglia, T., Chauveau, D., Hunter, D. R. & Young, D. S. mixtools: An R Package for Analyzing Mixture Models. *Journal of Statistical Software; Vol 1, Issue 6 (2010)*, doi:10.18637/jss.v032.i06 (2009).

14 Lötsch, J., Malkusch, S. & Ultsch, A. Comparative assessment of automated algorithms for the separation of one-dimensional Gaussian mixtures. *Informatics in Medicine Unlocked* **34**, 101113, doi:https://doi.org/10.1016/j.imu.2022.101113 (2022).

15 Bayes, M. & Price, M. An Essay towards Solving a Problem in the Doctrine of Chances. By the Late Rev. Mr. Bayes, F. R. S. Communicated by Mr. Price, in a Letter to John Canton, A. M. F. R. S. *Philosophical Transactions* **53**, 370-418, doi:10.1098/rstl.1763.0053 (1763).

16 Pearson, K. On the criterion that a given system of deviations from the probable in the case of a correlated system of variables is such that it can be reasonably supposed to have arisen from random sampling. *Philosophical Magazine, Series 5* **50**, 157-175 (1900).

17 Pearson, K. LIII. On lines and planes of closest fit to systems of points in space. *The London, Edinburgh, and Dublin Philosophical Magazine and Journal of Science* **2**, 559-572, doi:10.1080/14786440109462720 (1901).

18 Hyvärinen, A. & Oja, E. Independent component analysis: algorithms and applications. *Neural Networks* **13**, 411-430, doi:https://doi.org/10.1016/S0893-6080(00)00026-5 (2000).

19 Shepard, R. N. The analysis of proximities: Multidimensional scaling with an unknown distance function. II. *Psychometrika* **27**, 219-246 (1962).

20 Shepard, R. N. The analysis of proximities: multidimensional scaling with an unknown distance function. I. *Psychometrika* **27**, 125-140 (1962).

21 Tenenbaum, J. B., de Silva, V. & Langford, J. C. A global geometric framework for nonlinear dimensionality reduction. *Science* **290**, 2319-2323, doi:10.1126/science.290.5500.2319 (2000).

22 Van der Maaten, L. & Hinton, G. Visualizing Data using t-SNE. *J Machine Learn Res* **9**, 2579-2605 (2008).

23 Le, S., Josse, J. & Husson, F. c. FactoMineR: A Package for Multivariate Analysis. *Journal of Statistical Software* **25**, 1-18 (2008).

24 Marchini, J. L., Heaton, C. & Ripley, B. D. (2021).

25 Krijthe, J. H. (2015).

26 Venables, W. N. & Ripley, B. D. *Modern Applied Statistics with S*. (Springer, 2002).

27 Bartenhagen, C. (2022).

28 MacQueen, J. in *Proceedings of the Fifth Berkeley Symposium on Mathematical Statistics and Probability, Volume 1: Statistics.* 281-297 (University of California Press).

29 Kaufman, L. & Rousseeuw, P. J. Partitioning Around Medoids (Program PAM). *Finding Groups in Data*, 68-125, doi:https://doi.org/10.1002/9780470316801.ch2 (1990).

30 Ward Jr, J. H. Hierarchical grouping to optimize an objective function. *Journal of the American statistical association* **58**, 236-244 (1963).

31 Raymaekers, J. & Zamar, R. H. Pooled variable scaling for cluster analysis. *Bioinformatics* **36**, 3849-3855, doi:10.1093/bioinformatics/btaa243 (2020).

32 Maechler, M., Rousseeuw, P., Struyf, A., Hubert, M. & Hornik, K. cluster: Cluster Analysis Basics and Extensions. (2017).

33 Linderman, G. C. & Steinerberger, S. Clustering with t-SNE, Provably. *SIAM Journal on Mathematics of Data Science* **1**, 313-332, doi:10.1137/18M1216134 (2019).

34 Lötsch, J. & Ultsch, A. Current Projection Methods-Induced Biases at Subgroup Detection for Machine-Learning Based Data-Analysis of Biomedical Data. *International Journal of Molecular Sciences* **21**, doi:10.3390/ijms21010079 (2019).

35 R Development Core Team. R: A Language and Environment for Statistical Computing. (2008).

36 Wickham, H. *ggplot2: Elegant Graphics for Data Analysis*. (Springer-Verlag New York, 2009).

37 ggthemes: Extra Themes, Scales and Geoms for 'ggplot2' (2019).

38 R: A Language and Environment for Statistical Computing (Vienna, Austria, 2021).

39 ggforce: Accelerating 'ggplot2' (2020).

40 Gu, Z., Eils, R. & Schlesner, M. Complex heatmaps reveal patterns and correlations in multidimensional genomic data. *Bioinformatics* **32**, 2847-2849, doi:10.1093/bioinformatics/btw313 (2016).

Supplemental Figure 1: Results of censored likelihood multiple imputation of pain thresholds to cold and point pressure stimuli that had technical constraints at 0°C and 300 g of Frey hair, respectively. The plots show the probability density of the original data, the data from which the censored values were removed, and of the variables after imputation of the censored values. The x-scaling denotes the stimulus strength after zero-invariant log transformation ("sLog"). The figure has been created using the software package R (version 4.2.2 for Linux; https://CRAN.R-project.org/ [35]) and the R libraries "ggplot2" (https://cran.r-project.org/package=ggplot2 [36]).

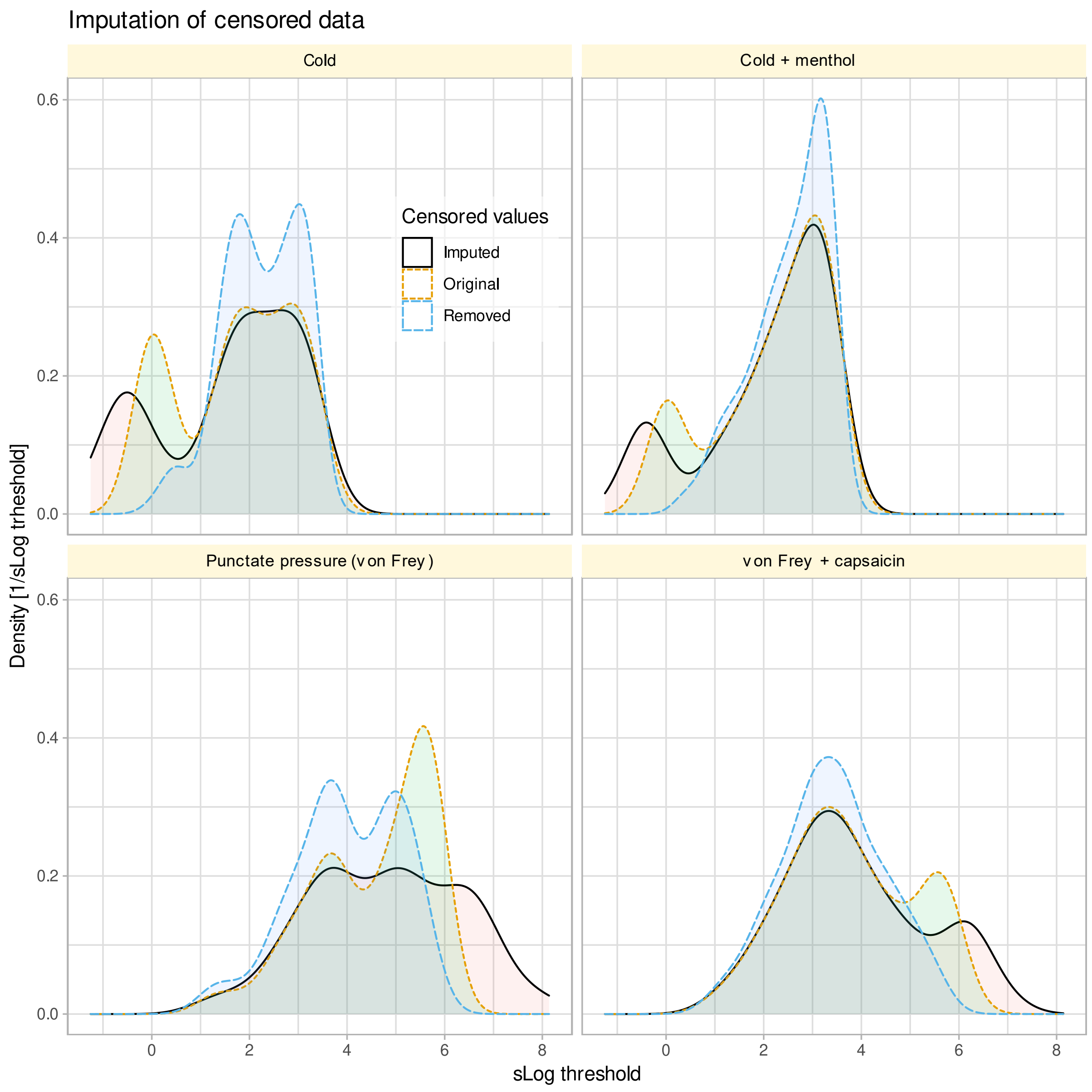

Supplemental Figure 2: Automated fits of Gaussian mixture models (GMM) to the single pain threshold variables. The figure shows the density distribution of the data as a grey line and as a histogram. A GMM was fitted to the data (dark blue line), with the number of modes of M automatically estimated by the appropriate algorithm. Estimated Bayesian boundaries between Gaussians are shown as magenta vertical lines, and the true boundaries according to the underlying model are indicated as blue dotted vertical lines. The figure was created using the software package R (version 4.2.0 for Linux; https://CRAN.R-project.org/ [35]) and or R package "opGMMassessment" (https://cran.r-project.org/package=opGMMassessment [14]).

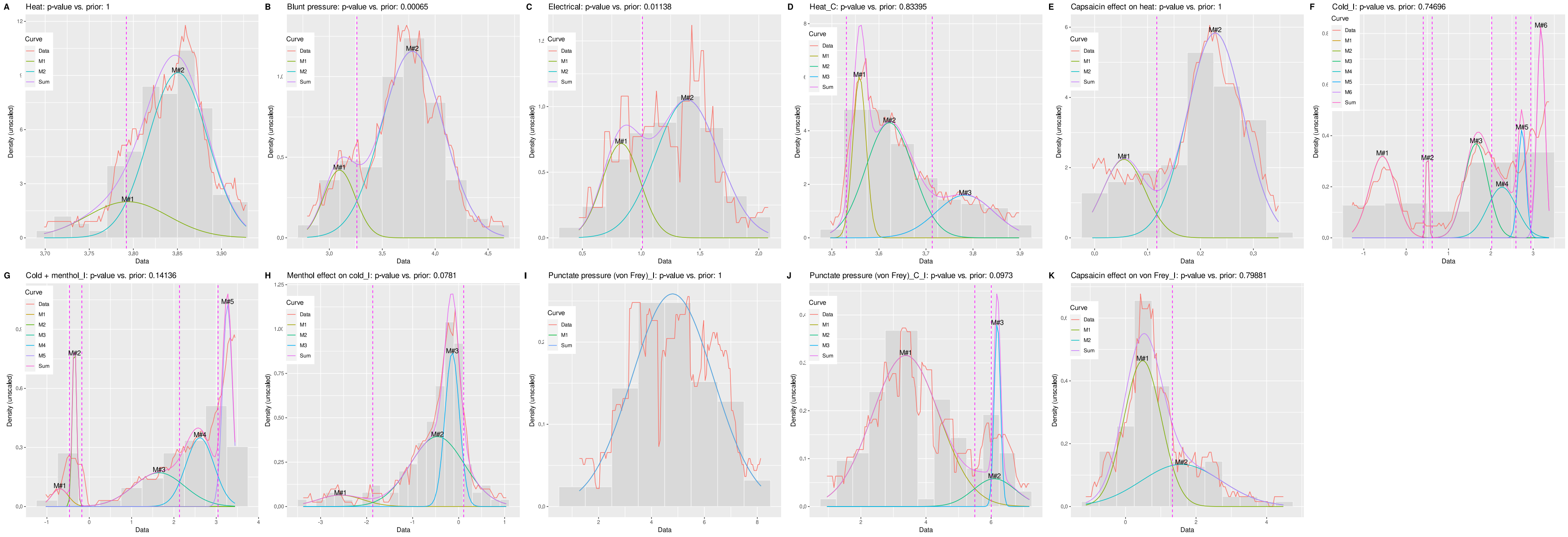

Supplemental Figure 3: Projections of pain thresholds using different methods (independent component analysis, ICA, isomap, multidimensional scaling, MDS, principal component analysis, PCA, and t-distributed stochastic neighborhood embedding, t-SNE). The single data are represented as dots of different colors and shapes according to their membership in the prior classes. The projection plane (dimension 2 versus dimension 1) is made of Voronoi cells around each data point, colored according to the membership of the respective data point to the prior classes, i.e., sexs. The coloring has been chosen from the “colorblind_pal” palette provided in the R library “ggthemes” (https://cran.r-project.org/package=ggthemes [37]).The figure has been created using the R software package (version 4.2.1 for Linux; https://CRAN.R-project.org/ [38]) and the R libraries “ggplot2” (https://cran.r-project.org/package=ggplot2 [36]) and “ggforce” (https://cran.r-project.org/package=ggforce [39]).

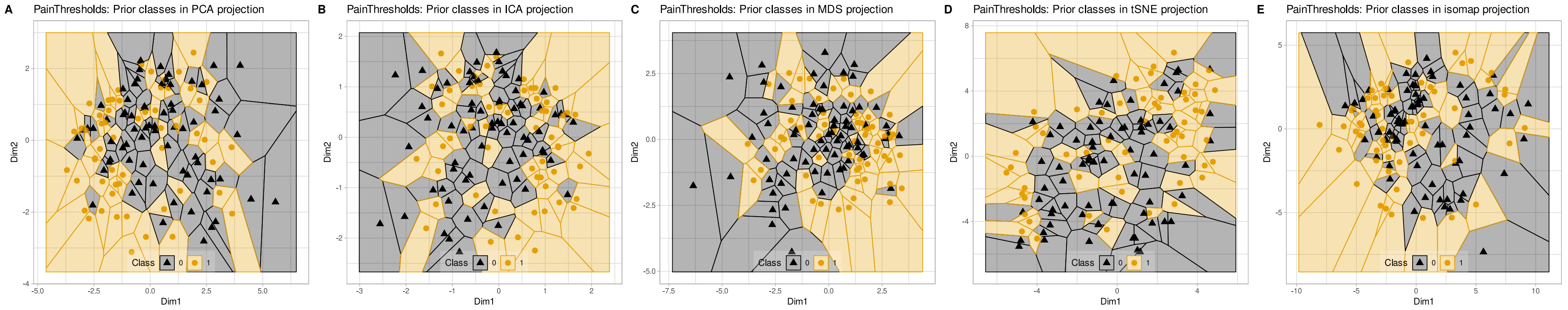

Supplemental Figure 4: Result of projection to the $\mathbb{R}^2$ plane followed by clustering of the pain thresholds. Projections were made using either principal component analysis (PCA) or t-distributed stochastic neighborhood embedding (t-SNE), and hierarchical clusters were determined using either Ward's linkage ("ward.D2") according to the corresponding command in the R programming language) or single linkage. The single data are represented as dots of different colors and shapes according to their membership in the prior classes. The projection plane (dimension 2 versus dimension 1) is made of Voronoi cells around each data point, colored according to the membership of the respective data point to the prior classes. The coloring has been chosen from the "colorblind_pal" palette provided in the R library "ggthemes" (https://cran.r-project.org/package=ggthemes [37]).The figure has been created using the R software package (version 4.2.1 for Linux; https://CRAN.R-project.org/ [38]) and the R libraries "ggplot2" (https://cran.r-project.org/package=ggplot2 [36]) and "ggforce" (https://cran.r-project.org/package=ggforce [39]).

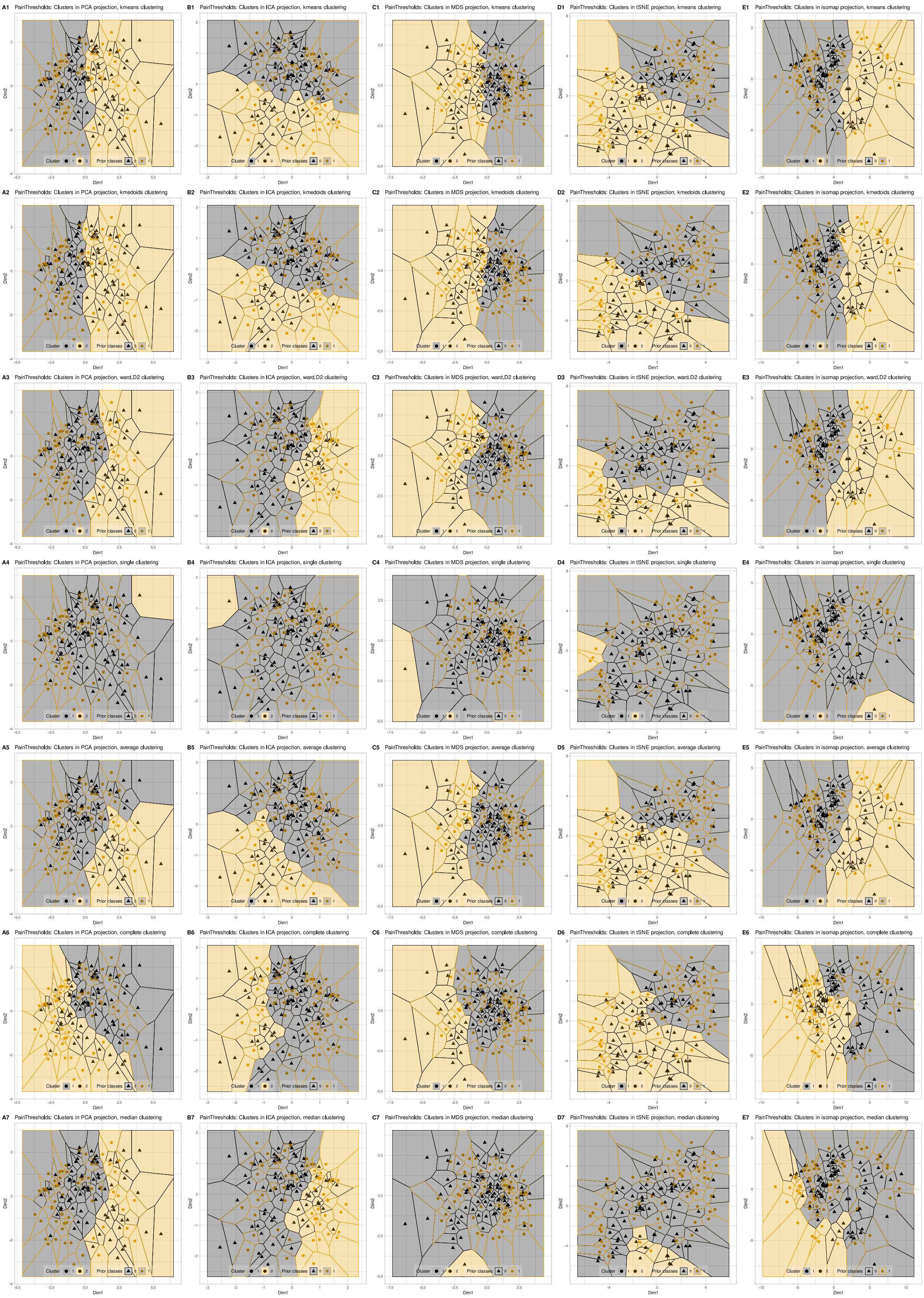
A1 PainThresholds: Clusters in PCA projection, kmeans clustering
B1 PainThresholds: Clusters in ICA projection, kmeans clustering
C1 PainThresholds: Clusters in MDS projection, kmeans clustering
D1 PainThresholds: Clusters in tSNE projection, kmeans clustering
E1 PainThresholds: Clusters in isomap projection, kmeans clustering
A2 PainThresholds: Clusters in PCA projection, kmedoids clustering
B2 PainThresholds: Clusters in ICA projection, kmedoids clustering
C2 PainThresholds: Clusters in MDS projection, kmedoids clustering
D2 PainThresholds: Clusters in tSNE projection, kmedoids clustering
E2 PainThresholds: Clusters in isomap projection, kmedoids clustering
A3 PainThresholds: Clusters in PCA projection, ward.D2 clustering
B3 PainThresholds: Clusters in ICA projection, ward.D2 clustering
C3 PainThresholds: Clusters in MDS projection, ward.D2 clustering
D3 PainThresholds: Clusters in tSNE projection, ward.D2 clustering
E3 PainThresholds: Clusters in isomap projection, ward.D2 clustering
A4 PainThresholds: Clusters in PCA projection, single clustering
B4 PainThresholds: Clusters in ICA projection, single clustering
C4 PainThresholds: Clusters in MDS projection, single clustering
D4 PainThresholds: Clusters in tSNE projection, single clustering
E4 PainThresholds: Clusters in isomap projection, single clustering
A5 PainThresholds: Clusters in PCA projection, average clustering
B5 PainThresholds: Clusters in ICA projection, average clustering
C5 PainThresholds: Clusters in MDS projection, average clustering
D5 PainThresholds: Clusters in tSNE projection, average clustering
E5 PainThresholds: Clusters in isomap projection, average clustering
A6 PainThresholds: Clusters in PCA projection, complete clustering
B6 PainThresholds: Clusters in ICA projection, complete clustering
C6 PainThresholds: Clusters in MDS projection, complete clustering
D6 PainThresholds: Clusters in tSNE projection, complete clustering
E6 PainThresholds: Clusters in isomap projection, complete clustering
A7 PainThresholds: Clusters in PCA projection, median clustering
B7 PainThresholds: Clusters in ICA projection, median clustering
C7 PainThresholds: Clusters in MDS projection, median clustering
D7 PainThresholds: Clusters in tSNE projection, median clustering
E7 PainThresholds: Clusters in isomap projection, median clustering
Dim1
Dim2
Cluster
Prior classes

Supplemental Figure 5: Performance of projection and clustering methods on the pain thresholds data sets, ranked by the p-values of chi square tests of cluster membership versus sex. **A:** Overall distribution of p values obtained during 100 cross-validation runs using boot resampling. **B:** Rank order of clustering results obtained with combinations of projection methods (independent component analysis, ICA, isomap, multidimensional scaling, MDS, principal component analysis, PCA, and t-distributed stochastic neighborhood embedding, t-SNE) and clustering algorithms (partitioning: k-means, k-medoids, hierarchical using single, Ward's, average, or complete linkage). The boxes show the 25th, 50th and 75th percentiles of ranks across data sets and cluster quality measures obtained in 100 repeated runs with a bootstrapping of the memberships to the clusters versus prior classes. Whiskers span the 95% confidence interval from the 2.5th to the 97.5th percentiles. Comparatively the best results were obtained with t-SNE, which is colored in darker blue to emphasize this method. The figure has been created using the software package R (version 4.2.1 for Linux; https://CRAN.R-project.org/ [35]) and the R libraries "ggplot2" (https://cran.r-project.org/package=ggplot2 [36]) and “ComplexHeatmap” (https://www.bioconductor.org/packages/ComplexHeatmap/ [40]).

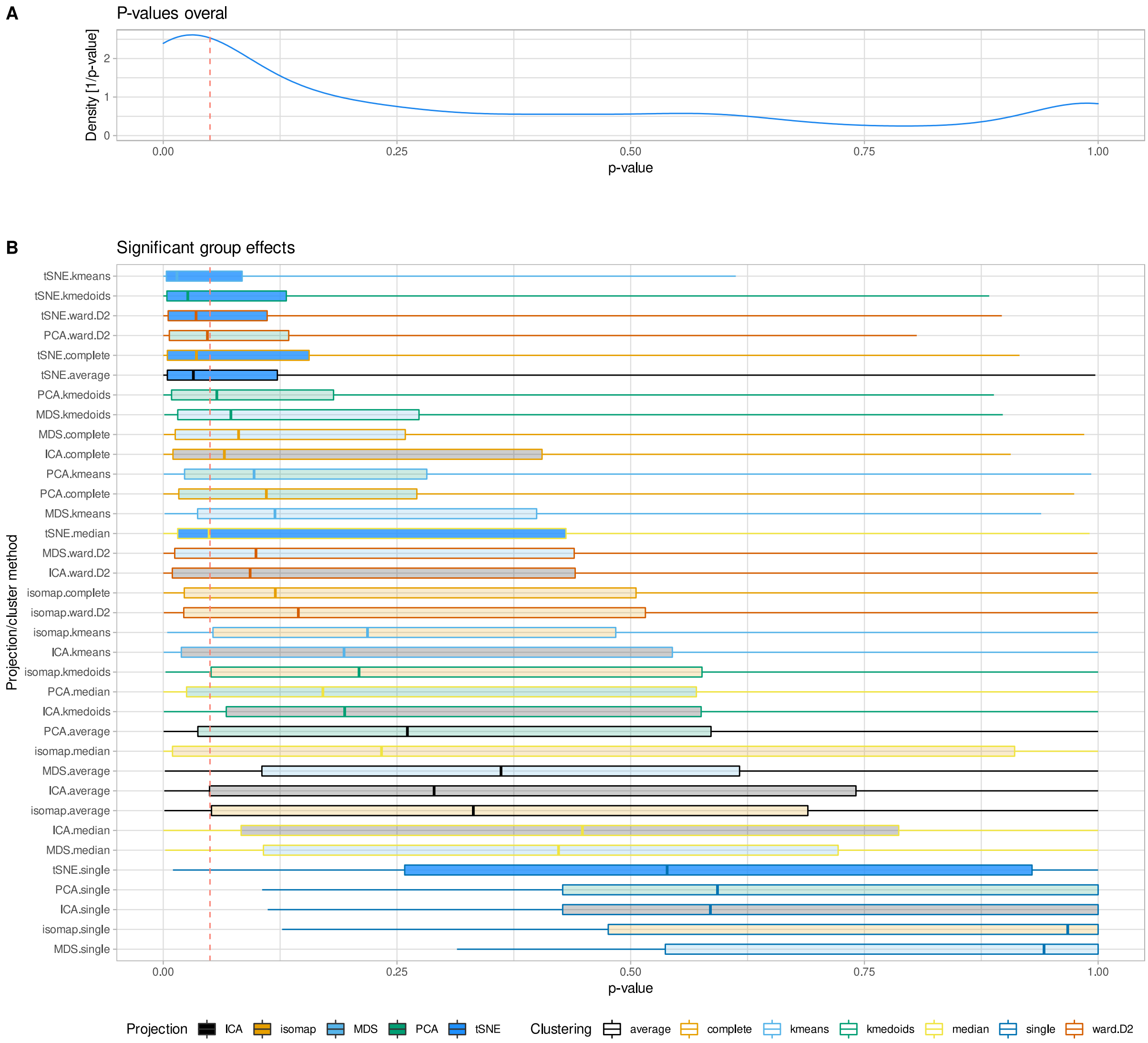
A
P-values overal
Density [1/p-value]
0
1
2
0.00
0.25
0.50
0.75
1.00
p-value
B
Significant group effects
Projection/cluster method
tSNE.kmeans
tSNE.kmedoids
tSNE.ward.D2
PCA.ward.D2
tSNE.complete
tSNE.average
PCA.kmedoids
MDS.kmedoids
MDS.complete
ICA.complete
PCA.kmeans
PCA.complete
MDS.kmeans
tSNE.median
MDS.ward.D2
ICA.ward.D2
isomap.complete
isomap.ward.D2
isomap.kmeans
ICA.kmeans
isomap.kmedoids
PCA.median
ICA.kmedoids
PCA.average
isomap.median
MDS.average
ICA.average
isomap.average
ICA.median
MDS.median
tSNE.single
PCA.single
ICA.single
isomap.single
MDS.single
0.00
0.25
0.50
0.75
1.00
p-value
Projection
ICA
isomap
MDS
PCA
tSNE
Clustering
average
complete
kmeans
kmedoids
median
single
ward.D2
